# Supplementary figures and images for: Accelerating the discovery of rare tree species in Amazonian forests: integrating long monitoring tree plot data with metabolomics and phylogenetics for the description of a new species in the hyperdiverse genus Inga Mill
Source: PeerJ. 2022 Aug 29;10:e13767. doi: 10.7717/peerj.13767 (PMC9435521; doi:10.7717/peerj.13767)

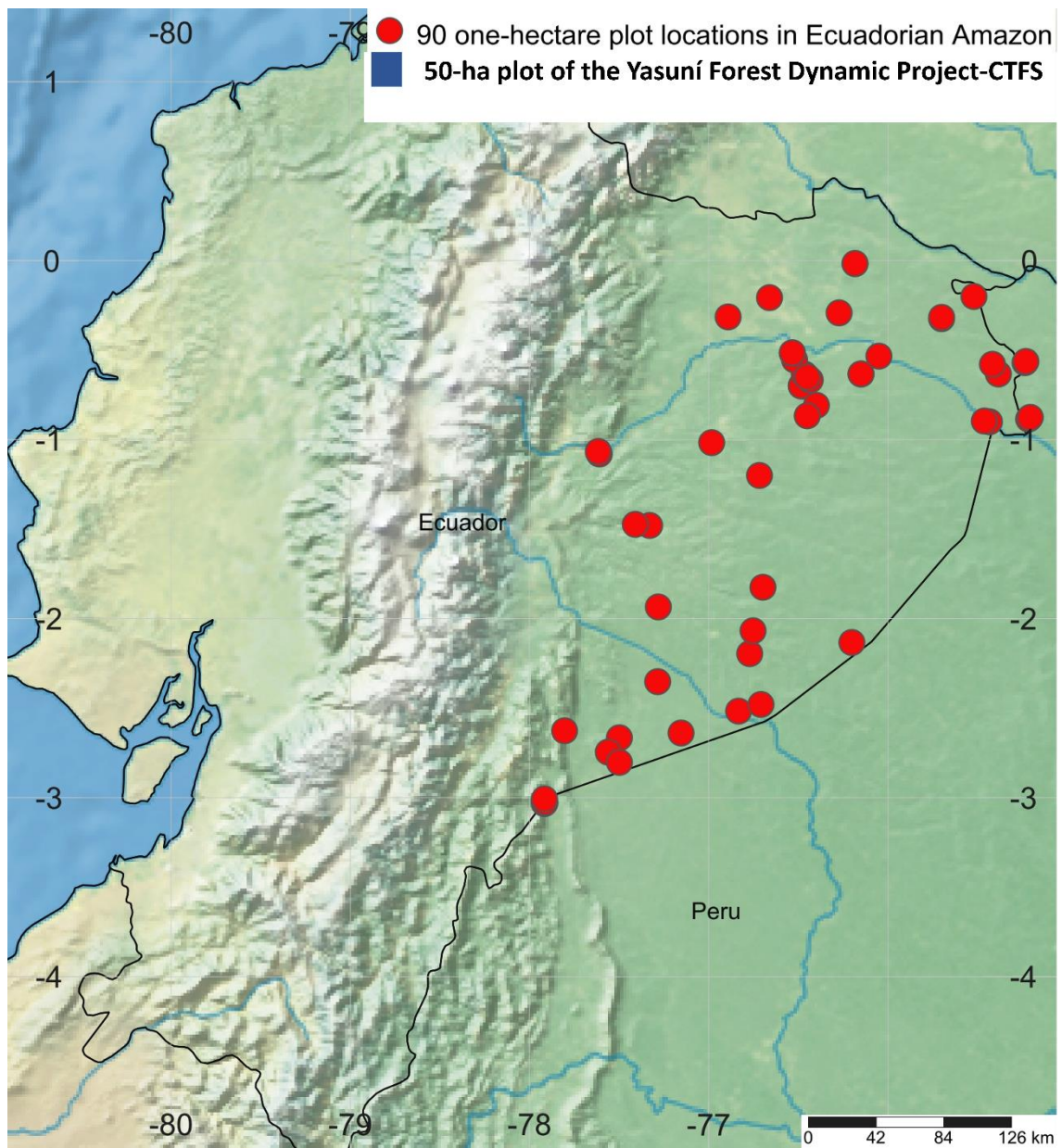

Supplement: Figure S1 — This plot network spans a longitudinal and latitudinal gradients in soils and climate, red dots corresponds to one-hectare plots meanwhile blue square corresponds to the Yasuní Forest Dynamic Project 50-hectare plot established by the Center for Tropical Forest Science from Smithsonian Institution. [file peerj-10-13767-s001.pdf]
